# BZR1 and BES1 transcription factors mediate brassinosteroid control over root system architecture in response to nitrogen availability

**DOI:** 10.3389/fpls.2024.1387321

**Published:** 2024-05-08

**Authors:** Mahamud Hossain Al-Mamun, Christopher Ian Cazzonelli, Priti Krishna

**Affiliations:** ^1^ School of Science, Western Sydney University, Richmond, NSW, Australia; ^2^ Hawkesbury Institute for the Environment, Western Sydney University, Richmond, NSW, Australia; ^3^ Faculty of Life Sciences, Graphic Era Deemed to be University, Dehradun, Uttarakhand, India

**Keywords:** brassinosteroid, root system architecture, nitrogen deficiency, BZR1, BES1, lateral roots

## Abstract

Plants modify their root system architecture (RSA) in response to nitrogen (N) deficiency. The plant steroidal hormone, brassinosteroid (BR), plays important roles in root growth and development. This study demonstrates that optimal levels of exogenous BR impact significant increases in lateral root length and numbers in *Arabidopsis* seedlings under mild N-deficient conditions as compared to untreated seedlings. The impact of BR on RSA was stronger under mild N deficiency than under N-sufficient conditions. The BR effects on RSA were mimicked in dominant mutants of BZR1 and BES1 (*bzr1-1D* and *bes1-D*) transcription factors, while the RSA was highly reduced in the BR-insensitive mutant *bri1-6*, confirming that BR signaling is essential for the development of RSA under both N-sufficient and N-deficient conditions. Exogenous BR and constitutive activity of BZR1 and BES1 in dominant mutants led to enhanced root meristem, meristematic cell number, and cortical cell length. Under mild N deficiency, *bzr1-1D* displayed higher fresh and dry shoot weights, chlorophyll content, and N levels in the shoot, as compared to the wild type. These results indicate that BR modulates RSA under both N-sufficient and N-deficient conditions via the transcription factors BES1/BZR1 module and confers tolerance to N deficiency.

## Highlight

- Brassinosteroid modulates the root system architecture in *Arabidopsis *via the BES1/BZR1 transcription factors module and confers tolerance to nitrogen deficiency.

## Introduction

The ability of plants to efficiently explore soil for water and minerals determines their competitive survival and productivity. Since roots are crucial for the perception and uptake of nutrients from the soil, the three-dimensional arrangement of the root system established by the length and thickness of the primary root (PR) and the density, length, thickness, and angles of lateral roots (LRs) of different orders determines the soil volume that can be explored by a dicotyledonous plant (Mounier et al., 2014; Kiba and Krapp, 2016). Additionally, the formation of root hairs allows plants to more intensively exploit the rhizosphere. This three-dimensional spatial arrangement of roots is referred to as root system architecture (RSA). Plant RSA is a highly dynamic system within which individual parts of the root system can be altered to achieve RSAs that are best suited to forage nutrients from different soil environments (Giehl and von Wirén, 2014; Giehl et al., 2014). The plasticity of RSA, guided by both genetic factors and environmental cues, is therefore crucial for plant performance, especially under suboptimal conditions in the root environment (Lynch, 1995; Malamy, 2005; Osmont et al., 2007).

Plants require nitrogen (N) in large amounts for their growth and survival; low availability of N in the soil can therefore be a major limiting factor for plant growth and development. The pool size of available N in soil changes over time due to uptake by plant roots, microbe-mediated cycling of N, and leaching of N (Lark et al., 2004; Kielland et al., 2007; Rothstein, 2009). Modulation of RSA in response to N availability is one of the most striking examples of developmental plasticity in plants in response to changing environmental conditions (Gruber et al., 2013). Plants continuously integrate N availability in the soil with their internal N demand and initiate adaptations in accordance with the extent of N limitation (Giehl and von Wirén, 2014). The status of N limitation is characterized by mild and severe deficiency. In the model plant *Arabidopsis*, mild and severe N deficiency can be distinguished by shoot fresh weight, shoot N concentration, and root growth. Mild deficiency (200–550 µM of externally supplied N) leads to decrease in shoot fresh weight and shoot N concentration but stimulation of root growth where LR emergence and PR and LR elongation increase (Gruber et al., 2013) but at the cost of a smaller root diameter (Qin et al., 2019). By contrast, severe N deficiency (<100 µM of externally supplied N) leads to growth inhibition of both PR and LR and a drop in shoot N concentration below the critical deficiency level (Gruber et al., 2013; Giehl et al., 2014). It is rationalized that under severe N deficiency, plants restrict root growth to economize the cost of development in favor of plant survival.

Phytohormones play key roles in plant adaptation to abiotic stresses, including stress imposed by N deficiency (Rubio et al., 2009; Krouk et al., 2010). Auxin plays an indispensable role in normal root development, and plants modulate their RSA in response to fluctuating soil environments by adjusting auxin distribution. Under mild N deficiency, auxin content decreases in the shoot (Avery et al., 1937; Avery and Pottorf, 1945) but increases in the root (Caba et al., 2000; Walch-Liu et al., 2006), resulting in increased PR length and LR emergence and elongation (Tian et al., 2008; Ma et al., 2014). The NITRATE TRANSPORTER1.1 (NRT1.1) serves as an integrator of nitrate signaling and auxin levels by functioning as an auxin transporter, promoting LR development under mild N deficiency, and suppressing the same under severe N deficiency (Krouk et al., 2010; Bouguyon et al., 2015; Meier et al., 2020).

Brassinosteroids (BRs) are an essential group of plant steroidal hormones that regulate numerous physiological and development processes, including root development (Vert et al., 2005; Clouse, 2011; Wei and Li, 2016; Li et al., 2020b). At low concentrations, BR promotes root growth, while at high concentrations, it inhibits root growth by controlling root meristem size (Müssig et al., 2000; González-García et al., 2011). BR signaling is triggered when BR binds to the cell surface receptor BRASSINOSTEROID INSENSITIVE 1 (BRI1), which leads to inactivation of BRASSINOSTEROID INSENSITIVE 2 (BIN2), a negative regulator of the two key transcription factors BZR1 and BES1, which are responsible for triggering BR responses (Clouse, 2002; Wang et al., 2002; Yin et al., 2002). In the absence of BR, BIN2 phosphorylates BZR1 and BES1, which leads to their degradation (Nam and Li, 2002; Kim et al., 2007). BR binding to BRI1, in addition to inactivating BIN2, initiates dephosphorylation by PROTEIN PHOSPHATASE 2A (PP2A) of BZR1 and BES1, leading to their activation and accumulation in the nucleus. Finally, active BZR1 and BES1 bind to DNA to regulate the expression of BR-response genes (He et al., 2005; Yin et al., 2005; Sun et al., 2010; Yu et al., 2011). BZR1 and BES1 are homologous transcription factors with redundant as well as unique roles (Wang et al., 2002; Yin et al., 2002, 2005). Dominant mutations in BZR1 and BES1 (*bzr1-1D* and *bes1-D*) promote a hypophosphorylated state of the protein, resulting in constitutive activity and, consequentially, constitutive BR responses (Wang et al., 2002; Yin et al., 2002; Tang et al., 2011). The constitutive activity of BZR1 and BES1 also confers resistance to the compound brassinazole (BRZ), a specific inhibitor of the BR biosynthesis enzyme DWARF4 (DWF4) (Asami et al., 2001). Both BES1 and BZR1 are involved in the maintenance of root apical meristem activities (Lee et al., 2015). In rice, BZR1 activity is positively correlated with LR formation (Hou et al., 2022), and in *Arabidopsis*, BES1-regulated XYLOGLUCAN ENDOTRANSGLUCOSYLASE 19 (*XTH19*) and *XTH23* genes are involved in LR development under salt stress (Xu et al., 2020). However, in general, information on the roles of BES1 and BZR1 in modulating RSA under stress is limited.

Recent studies demonstrate that BR modulates PR elongation under mild N deficiency, which upregulates BR biosynthesis genes (Jia et al., 2020) and promotes BR signaling (Jia et al., 2019). A BR-auxin hormonal module has been uncovered recently, which emphasizes the importance of crosstalk between the two hormones in synergistically stimulating cell elongation under mild N deficiency (Jia et al., 2021). While these studies point toward the role of BR in N deficiency, a more comprehensive account of how BR impacts RSA and how the changes are correlated with plant performance is lacking. We show here that exogenous application of BR induces changes in the RSA of *Arabidopsis* seedlings under both N-sufficient and N-deficient conditions and that the RSA changes in the gain-of-function BR mutants, *bzr1-1D* and *bes1-D*, are consistent with those induced by exogenous BR. The *bzr1-1D* showed enhancement of all growth traits measured, indicating that constitutive BR signaling provides an advantage to *Arabidopsis* seedlings under N-deficient conditions. These results indicate that BR modulates RSA via the BES1/BZR1 module and that the RSA changes correlate with higher shoot N content, suggesting that BR plays a role in conferring tolerance to N deficiency in *Arabidopsis*.

## Research methods

### Plant materials

Six *Arabidopsis* genotypes were used in this study: *Arabidopsis thaliana* ecotypes Columbia (Col-0), Enkheim-1 (En-1) and En-2, gain-of-function mutants *bzr1-1D* (Wang et al., 2002) and *bes1-D* (Yin et al., 2002), and *bri1-6*, a weak mutant allele of BRI1 (Noguchi et al., 1999). The accessions Col-0, En-1, and En-2 were used as background for *bzr1-1D*, *bes1-D*, and *bri1-6* mutants, respectively. The lines Col-0 (CS76113), En-1 (CS1136), En-2 (CS1138), *bes1-D* (CS65988), and *bri1-6* (CS399) were purchased from the Arabidopsis Biological Resource Centre (ABRC, USA), and *bzr1-1D* seeds were collected from Dr Zhiyong Wang’s Laboratory, Carnegie Institution for Science, USA.

### *Arabidopsis* growth

Surface sterilized seeds as per the method of Kawa et al. (2016) were germinated in the dark at 4°C for 72 hours on one-half strength Murashige and Skoog (MS) medium (Murashige and Skoog, 1962) supplemented with 1% sucrose, 0.1% MES monohydrate, 0.8% Bacto agar, and 0.1% Gamborg’s vitamin (G1019; Sigma-Aldrich, St. Louis, MO, USA). The plates were transferred to and placed vertically in a Conviron A1000 growth cabinet maintained at 22°C with a light intensity of 100 μmol m^−2^ s^−1^ and a 16/8-hour light/dark cycle.

Three-day-old, similar-sized seedlings were transferred to MS plates (five seedlings per plate) for different treatments and further grown for 9 days. The plates were placed in a random manner in the growth chamber, and their position was changed on alternate days to avoid position effects on seedling growth. N was supplied in MS basal salt without N (MSP10; Caisson Labs, Smithfield, UT, USA) as either full N (11,400 μM) or a range of low N concentrations (10 μM, 100 μM, 275 μM, and 550 μM). For full N, 1,000 μM N from ammonium nitrate (NH_4_NO_3_) and 9,400 μM N from potassium nitrate (KNO_3_) were added to the MS basal salt (without N), and for low N concentrations, only KNO_3_ was supplied (Gruber et al., 2013). The osmotic potential of these media was maintained by adding KCL. For BR treatment, MS medium was supplemented with 0.05 nM 24-epibrassinolide (EBR), if not indicated otherwise, and for BRZ treatment, plates were supplied with 2 μM BRZ. Stock solutions of EBR (CAS no. 78821-43-9; Sigma) at 20 mM and those of BRZ (CAS no. 280129-83-1, Sigma) at 20 μM were prepared using pure ethanol (Sigma) and dimethyl sulfoxide (DMSO) (Sigma), respectively. When supplementing the MS medium with EBR or BRZ, an identical volume of the solvent was added to the controls (Singh et al., 2014; Krishna et al., 2017). All experiments were repeated at least twice.

### Root phenotyping analysis

Nine days after seedling transfer, the MS plates were scanned using an EPSON Expression 11000XL scanner in color at 600 dots per inch (dpi) resolution. Scanned images were analyzed using the EZ-Rhizo II software (Armengaud et al., 2009). Seedlings that were damaged during transfer were excluded from the analysis. The total root length was calculated for each plant by summing the PR and LR lengths. The average LR length was calculated by dividing the total LR length by the number of LRs, and the number of LR per cm was calculated by dividing the total number of LR by the PR length (Gruber et al., 2013).

### Shoot and root fresh and dry weight

Seedlings grown on MS medium for 3 days were transferred to treatment plates and allowed to grow for 13 days. Shoots were harvested in Eppendorf tubes and weighed for fresh weight. Roots were gently pulled out from the MS plates, and any medium sticking to the roots was carefully removed. After a gentle press between Kimwipes, roots were weighed and stored in Eppendorf tubes. The root and shoot samples were dried at 60°C in an oven until constant weight was reached.

### Root meristem size measurements

For root meristem size and cortical cell length measurements, roots were stained with propidium iodide (10 µg/mL) for 2 min, followed by three washes with distilled water. Root meristem images were taken using a laser scanning confocal microscope (Leica TCS SP5 MP), controlled by the LAS AF software (Leica Microsystems, Wetzlar, Germany). Propidium iodide was viewed at an excitation wavelength of 488 nm with emission collected at 575 nm. Root meristem size was defined as the distance from the quiescent center (QC) to the first cells displaying elongation (Cazzonelli et al., 2013).

### Chlorophyll measurements

Leaf samples weighing approximately 8–10 mg were harvested and placed in an Eppendorf tube. To this, 1 mL of 80% acetone was added, and the mixture was incubated at room temperature for 48 hours. Chlorophyll *a* (Chl-*a*), total chlorophylls (Chl-*a*+*b*), and total carotenoids (Cc) were measured at 470 nm, 645 nm, and 652 nm, respectively, using a spectrophotometer (SPECTROstar^©^ *Nano*). Chl-*a*, Chl-*a*+b, and Cc concentrations were calculated as described by Lichtenthaler and Wellburn (1983), and the pigment content was expressed as mg/g fresh weight.

### Elemental assay

Shoots and roots were harvested from plants grown for 13 days in full N (11,400 µM) and under mild N deficiency (550 µM). After drying at 60°C for 72 hours, the samples were milled to a fine powder using TissueLyser II^®^ (QIAGEN, Hilden, Germany) for 2 min at 20-Hz speed, using stainless steel beads (~3 mm in diameter) in a 2-mL microtube. Approximately 6 mg of each sample was used for the analysis of C and N using the LECO TruMac CN analyzer (LECO Corporation, St. Joseph, MI, USA).

### RNA isolation and qPCR

Roots of seedlings grown for 4 days either in full N (11,400 µM) or under mild N deficiency (550 µM) were harvested, quickly frozen in liquid nitrogen, and physically disrupted using needles in Eppendorf tubes. Total RNA was extracted using the RNeasy Plant Mini Kit (QIAGEN, Valencia, CA, USA). Following quantification with NanoDrop ND-2000^®^ (Thermo Scientific, Waltham, MA, USA), 1 µg of RNA was transcribed into cDNA with QuantiNova Reverse Transcription Kit (QIAGEN, Valencia, CA, USA). qPCRs were performed using QuantiNova SYBR green PCR Kit (QIAGEN, Valencia, CA, USA) with Rotor-Gene^®^ Q (QIAGEN, Hilden, Germany) using the gene-specific primers listed in **Supplementary Table S1**. Gene-specific primer pairs were designed using the Primer3Plus online software (http://primer3plus.com/) (Untergasser et al., 2012). The cycling conditions were 5 min at 95°C, followed by 40 cycles of 5 s at 95°C and 10 s at 60°C. The relative expression of the tested genes was normalized against the *Arabidopsis U-box* gene. Relative expression values were calculated using the 2^−ΔΔCT^ method (Schmittgen and Livak, 2008). Three biological replications, each with three technical repetitions, were performed for all reactions.

### Statistical analysis and data visualization

All growth and stress response data, unless stated otherwise, were analyzed and visualized using the Systat software SigmaPlot (version 14.0). The significance of differences was analyzed by two-way analysis of variance (ANOVA). Comparison among treatment means was conducted using Tukey’s multiple-comparison test.

## Results

### BR-mediated changes in RSA under nitrogen-sufficient and nitrogen-deficient conditions are concentration-dependent

**Figure 1:**
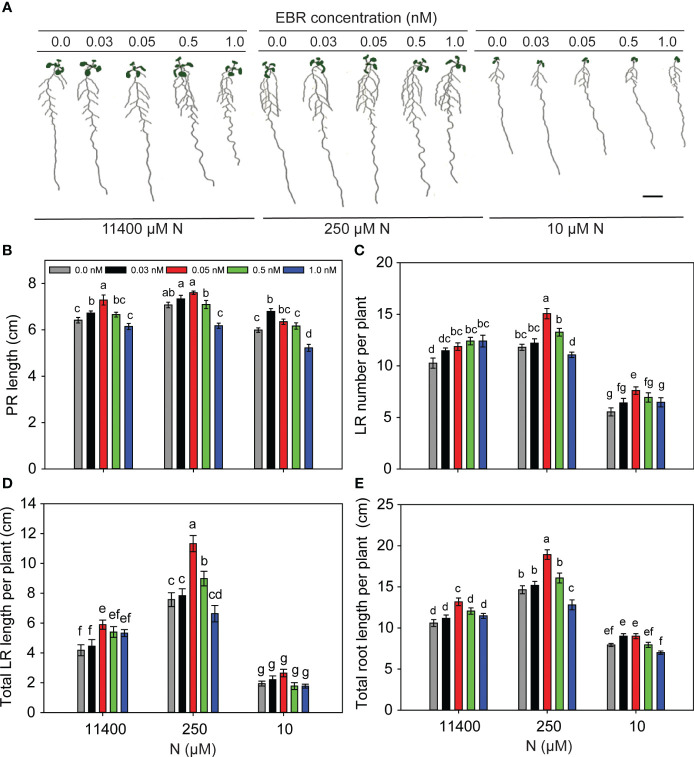
BR concentration-mediated changes to RSA under N-sufficient and N-deficient conditions. Seedlings grown in full N for 3 days were transferred to N-sufficient (11,400 µM N) and N-deficient (250 µM and 10 µM N) media supplemented with a range of EBR concentrations (0.0 nM, 0.03 nM, 0.05 nM, 0.5 nM, and 1.0 nM). Root morphology was analyzed at 9 DAT. Representative images of plants of two independent experiments **(A)**, PR length (cm) **(B)**, LR number per plant **(C)**, total LR length per plant (cm) **(D)**, and total root length per plant (cm) **(E)**. Bars represent means ± SEM (*n* = 15). Different letters indicate significant differences at *p* < 0.05 according to two-way ANOVA and Tukey’s multiple-comparison test by SigmaPlot. Scale bar, 1 cm. BR, brassinosteroid; RSA, root system architecture; EBR, 24-epibrassinolide; DAT, days after transfer; PR, primary root; LR, lateral root.

It is known that low concentrations of BR promote while high concentrations inhibit root growth (Müssig et al., 2003; Jantapo et al., 2021). To see the effect of BR levels on RSA of *Arabidopsis* seedlings under different N conditions, 3-day-old seedlings were transferred to treatment plates supplemented with 0 nM, 0.03 nM, 0.05 nM, 0.5 nM, and 1.0 nM EBR under N-sufficient (11,400 µM) and N-deficient (250 µM and 10 µM) conditions. Since enhanced root growth promotes exploration and utilization of nutrients, plates were scanned and analyzed for root traits (PR length, LR number/plant, total LR length/plant, and total root length/plant) 8 days after transfer (DAT) (**Figures 1A–E**). PR lengths increased with EBR concentrations of 0.03–0.05 nM, declining thereafter with 1 nM EBR being inhibitory as compared to the untreated control (**Figures 1A, B**). EBR at 0.05 nM promoted the highest increase in LR number (28%, **Figure 1C**), total LR length (50%, **Figure 1D**), and total root length (29%, **Figure 1E**) per plant, as compared to the control (no BR), under mild (250 µM) N deficiency. EBR also induced a significant increase in LR number (37% as compared to the untreated control) under severe N deficiency (**Figure 1C**), indicating that EBR directly or indirectly is a major regulator of LR formation in *Arabidopsis*. In the absence of EBR (0 nM), mild N deficiency (250 µM) stimulated all traits of the RSA measured, while severe N deficiency (10 µM) suppressed most of these traits (**Figures 1B, D**). These results are consistent with previous observations of enhanced PR and total LR lengths (foraging response) under mild N deficiency (López-Bucio et al., 2003) and restriction of growth as a survival strategy under severe N deficiency (Gruber et al., 2013). The novelty of our results lies in the observation that exogenous EBR at an optimal concentration of 0.05 nM positively modulated root growth in *Arabidopsis* seedlings under N starvation over and above that induced by N starvation alone, with maximal BR effects occurring under mild N deficiency (**Figures 1C, E**).

### BR impacts RSA under a range of N conditions

**Figure 2:**
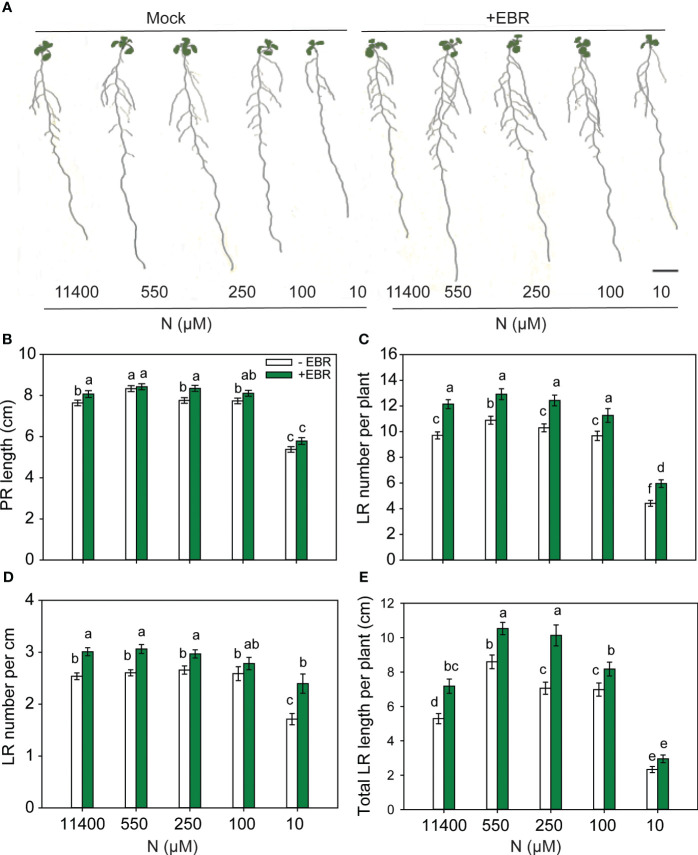
BR-mediated changes to RSA under different N conditions. Seedlings grown in full N for 3 days were transferred to N-sufficient (11,400 µM N) and N-deficient (550 µM, 250 µM, 100 µM, and 10 µM N) media supplemented with 0.05 nM EBR. Root morphology was analyzed at 9 DAT. Representative images of plant **(A)**, PR length (cm) **(B)**, LR number per plant **(C)**, LR number per cm **(D)**, and total LR length per plant (cm) **(E)**. Bars represent means ± SEM (two independent biological replicates, *n* = 30). Different letters indicate significant differences at *p* < 0.05 according to two-way ANOVA and Tukey’s multiple-comparison test by SigmaPlot. Scale bar, 1 cm. BR, brassinosteroid; RSA, root system architecture; EBR, 24-epibrassinolide; DAT, days after transfer; PR, primary root; LR, lateral root.

To test the effect of exogenous EBR at the optimal concentration of 0.05 nM under a range of N supply conditions (11,400 µM, 550 µM, 250 µM, 100 µM, and 10 µM), 3-day-old seedlings grown on MS medium were transferred to plates with different N amounts and allowed to grow for 9 days before root traits were measured. PR length decreased in response to severe N deficiency as compared to other N conditions, and treatment with EBR caused only minor increases in the PR lengths of seedlings (**Figures 2A, B**). Consistent with the results represented in **Figure 1C**, EBR enhanced LR number per plant at all concentrations of N (**Figure 2C**). EBR increased total LR length across all concentrations of N with the greatest effect being at 250 µM N (44% increase over untreated seedlings) (**Figure 2E**). Although EBR enhanced root traits, mainly the number and length of LR, untreated seedlings also showed a foraging response between 11,400–100 µm N and growth retardation at 10 µM N. Based on these results, it can be concluded that 1) RSA follows a foraging response between 550 and 100 µM N (mild N deficiency) in both untreated and EBR-treated seedlings, 2) EBR effect is most prominent on LR length and LR number, and 3) under severe N deficiency (10 µM N), EBR increases LR number but has an insignificant effect on LR elongation (length).

### Constitutive activity of BZR1 and BES1 enhances root foraging traits under N starvation

**Figure 3:**
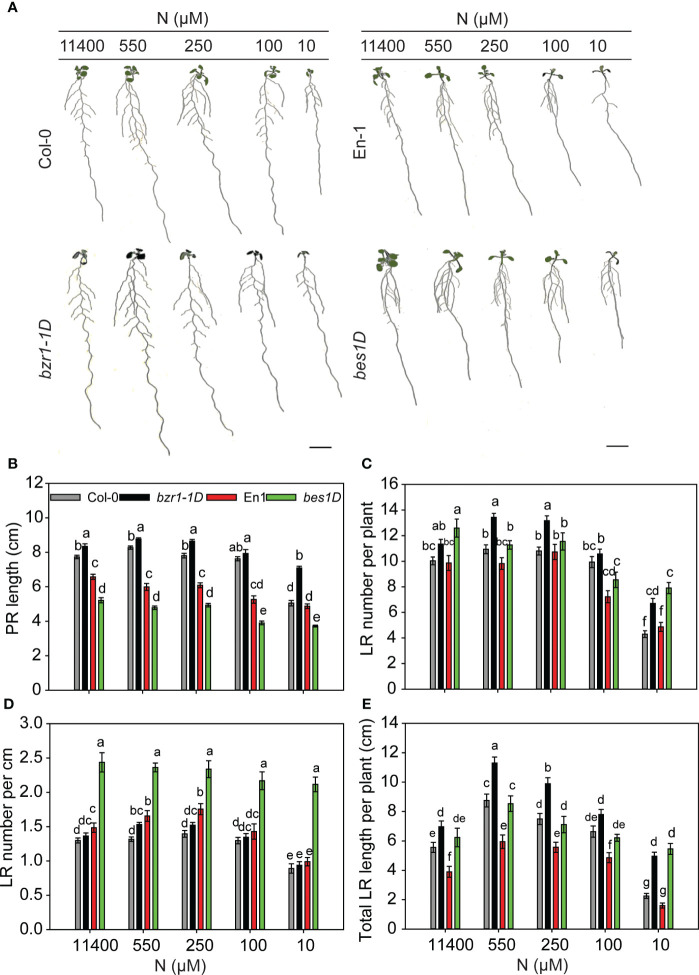
RSA changes in gain-of-function mutants *bzr1-1D* and *bes1-D* and their respective WTs under different N conditions. Seedlings grown in full N for 3 days were transferred to N-sufficient (11,400 µM N) and N-deficient (550 µM, 250 µM, 100 µM, and 10 µM N) media. Root morphology was analyzed at 9 DAT. Representative images of plant **(A)**, PR length (cm) **(B)**, LR number per plant **(C)**, LR number per cm **(D)**, and total LR length per plant (cm) **(E)**. Bars represent means ± SEM (two independent biological replicates, *n* = 30). Different letters indicate significant differences at *p* < 0.05 according to two-way ANOVA and Tukey’s multiple-comparison test by SigmaPlot. Scale bar, 1 cm. RSA, root system architecture; WTs, wild types; DAT, days after transfer; PR, primary root; LR, lateral root.

To see whether BR-mediated RSA changes are facilitated by the BES1/BZR1 module, we used the gain-of-function mutants *bzr1-1D* and *bes1-D* and their corresponding wild types (WTs) Col-0 and En-1, respectively. The general trend in RSA changes over the N concentrations used, i.e., an increase in PR length and LR number and length under mild N deficiency and a significant reduction under severe N deficiency, was conserved in untreated and EBR-treated WT (**Figure 2**) and *bzr1-1D* and *bes1-D* seedlings with the exception of PR length in *bes1-D* (**Figure 3**). A remarkable difference was observed in the PR lengths of *bzr1-1D* and *bes1-D* from their WTs (**Figures 3A, B**). *bzr1-1D* produced longer PR than its corresponding WT Col-0 under both N-sufficient (8% increase at 11,400 µM N) and N-deficient (6%, 11%, and 40% increase at 550 µM, 250 µM, and 10 µM N, respectively) conditions. By contrast, *bes1-D* produced shorter PR than its corresponding WT En-1 (**Figures 3A, B**). The reduction in PR length of *bes1-D* seedlings, as compared to WT, ranged from 25% to 31% at 11,400 µM to 10 µM N, respectively (**Figure 3B**). The LR number and length were increased to varying extents in both *bzr1-1D* and *bes1-D* seedlings under all N conditions as compared to their respective WTs, with the maximum relative increase occurring at 10 µM N (severe deficiency) (**Figures 3C, E**). For example, the number of LRs in *bzr1-1D* increased by 13%, 233%, 223%, and 56% at 11,400 µM, 550 µM, 250 µM, and 10 µM N, respectively, and the total LR length in *bzr1-1D* and *bes1-D* seedlings was increased over WT by 120% and 241%, respectively, at 10 µM N. A noteworthy observation is that the LR density in *bes1-D* seedlings was significantly higher than its WT at all N concentrations (64%, 43%, 33%, 51%, and 114% increase at 11,400 µM, 550 µM, 250 µM, 100 µM, and 10 µM N, respectively) and also considerably higher than in *bzr1-1D* (**Figure 3D**). This is due to the shorter PR length and increased LR number per plant in *bes1-D*. The consistent enhancement of root traits by exogenous EBR and by constitutive transcription factor activity of BZR1 and BES1 in *bzr1-1D* and *bes1-D*, respectively, indicates that BR-mediated RSA under different N conditions occurs via the BZR1/BES1 module in *Arabidopsis*. Our results also reveal that BES1 and BZR1 differentially influence the LR and PR features of *Arabidopsis* RSA.

### *bzr1-1D* is more resistant to BR biosynthesis inhibitor BRZ across N treatments

**Figure 4:**
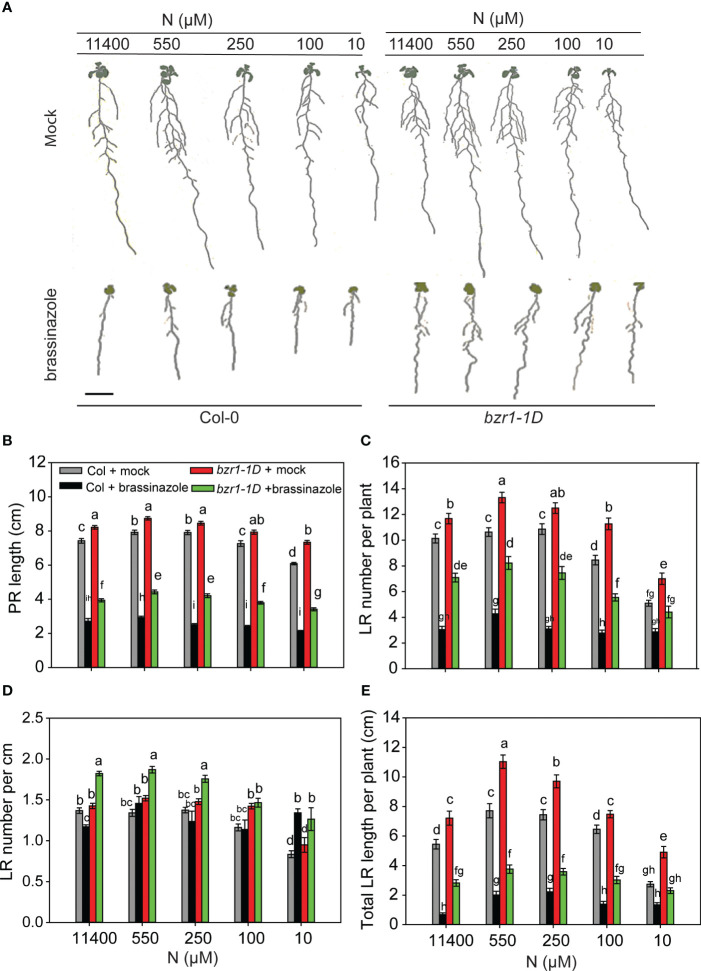
BRZ effect on RSA in wild type (Col-0) and *bzr1-1D* under different N conditions. Seedlings grown in full N for 3 days were transferred to N-sufficient (11,400 μM) and N-deficient (550 µM, 250 µM, 100 µM, and 10 µM N) media supplemented with 2 µM BRZ. Root morphology was analyzed at 9 DAT. Representative images of plant **(A)**, PR length (cm) **(B)**, LR number per plant **(C)**, LR number per cm **(D)**, and total LR length per plant (cm) **(E)**. Bars represent means ± SEM (two independent biological replicates, *n* = 22). Different letters indicate significant differences at *p* < 0.05 according to two-way ANOVA and Tukey’s multiple-comparison test by SigmaPlot. Scale bar, 1 cm. BRZ, brassinazole; RSA, root system architecture; DAT, days after transfer; PR, primary root; LR, lateral root.

Treatment of *Arabidopsis* seedlings with 2 µM BRZ, a BR biosynthesis inhibitor, severely arrested PR length, LR number, and LR length in WT Col-0 at all N conditions (**Figures 4A–C, E**). BRZ also inhibited RSA traits in *bzr1-1D* but not to the same extent as in WT (**Figures 4A–C, E**). For example, PR length in WT was reduced ~ 3-fold in WT by BRZ, whereas in *bzr1-1D*, the reduction was ~2-fold across N conditions (**Figure 4B**). Similarly, BRZ decreased LR number in WT by 3.3-, 2.4-, 3.6-, 3-, and 1.7-fold at 11,400 µM, 550 µM, 250 µM, 100 µM, and 10 µM N supply, respectively, whereas in *bzr1-1D*, there was ~1.6-fold reduction in LR number across different N conditions (**Figure 4C**). The LR density was the highest in *bzr1-1D* treated with BRZ under N-sufficient and mild N-deficient conditions (**Figure 4D**). Total LR length was decreased with BRZ in both WT and *bzr1-1D* (**Figure 4E**), but in comparison with WT, *bzr1-1D* produced ~2 fold more LRs and greater LR length in the presence of BRZ. These results indicate that while *bzr1-1D* is more resistant to BRZ, BR biosynthesis is essential for optimal root growth even with the constitutive activity of BZR1 in the *bzr1-1D* mutant.

### *bes1-D* is more resistant to BRZ than WT across N treatments

**Figure 5:**
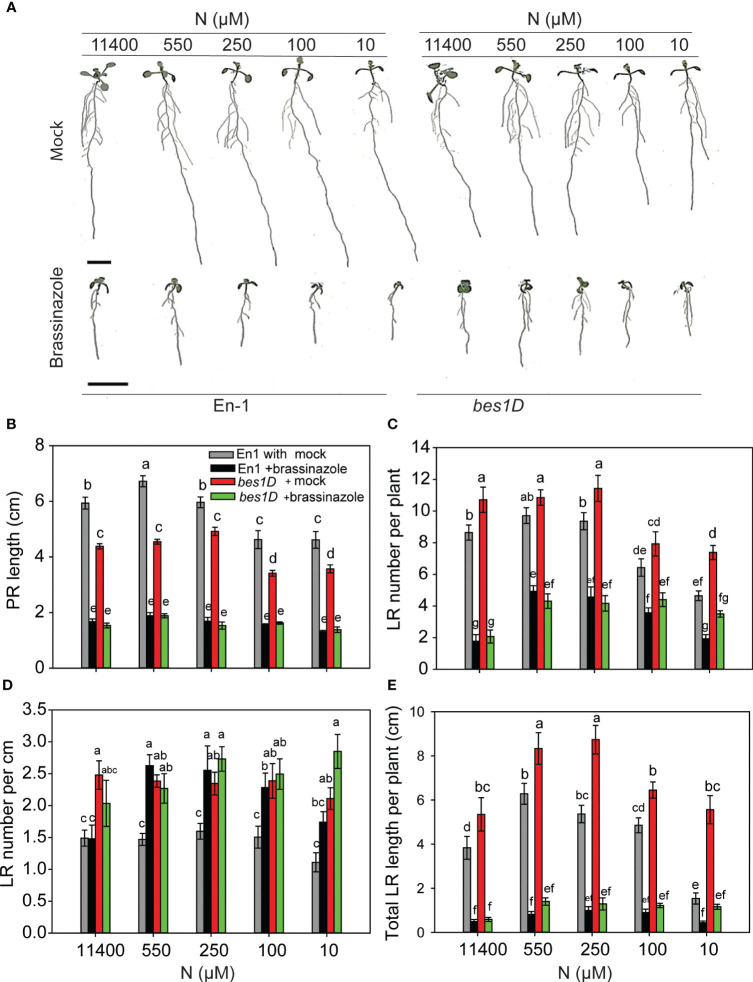
BRZ effect on RSA in wild type (En-1) and *bes1-D* under different N conditions. Seedlings grown in full N for 3 days were transferred to N-sufficient (11,400 μM) and N-deficient (550 µM, 250 µM, 100 µM, and 10 µM N) media supplemented with 2 µM BRZ. Root morphology was analyzed at 9 DAT. Representative images of plant **(A)**, PR length (cm) **(B)**, LR number per plant **(C)**, LR number per cm **(D)**, and total LR length per plant (cm) **(E)**. Bars represent means ± SEM (two independent biological replicates, *n* = 22). Different letters indicate significant differences at *p* < 0.05 according to two-way ANOVA and Tukey’s multiple-comparison test by SigmaPlot. Scale bar, 1 cm. BRZ, brassinazole; RSA, root system architecture; DAT, days after transfer; PR, primary root; LR, lateral root.

BRZ inhibited RSA traits in both WT and *bes1-D* across different N conditions. *bes1-D* showed more resistance to a reduction in PR length (**Figures 5A, B**) and was more sensitive to BRZ than its corresponding WT for the reduction in LR number per plant and total LR length (**Figures 5C, E**). While BRZ reduced LR number by ~5-fold in both WT and *bes1-D* at full N (11,400 µM), this effect was less under lower N conditions. For example, LR numbers were reduced 2- and 2.5-fold in WT and *bes1-D*, respectively, at 550 µM N supply as compared to the mock (**Figure 5C**). Consequently, LR density also increased under low N conditions for WT (**Figure 5D**). These results indicate that while the constitutive activity of BES1 in *bes1-D* positively impacts LR number and length, these effects are dependent on optimal endogenous BR levels, which may in turn impact other signaling pathways involved in root development.

### *bri1-6* is moderately responsive to N deficiency

**Figure 6:**
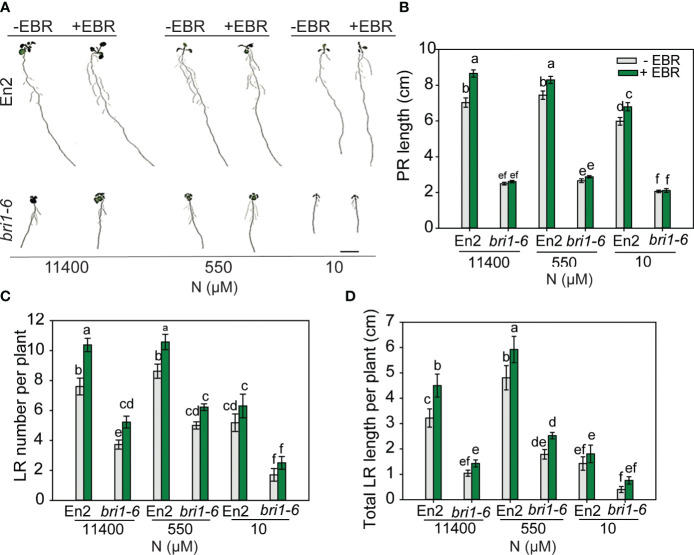
RSA changes in wild type (En-2) and bri1-6 under different N conditions. Seedlings grown in full N for 3 days were transferred to N-sufficient (11,400 μM) and N-deficient (550 µM and 10 µM N) media supplemented with 0.05 nM EBR. Root morphology was analyzed at 9 DAT. Representative images of plant **(A)**, PR length (cm) **(B)**, LR number per plant **(C)**, and total LR length per plant (cm) **(D)**. Bars represent means ± SEM (two independent biological replicates, *n* = 22). Different letters indicate significant differences at *p* < 0.05 according to two-way ANOVA and Tukey’s multiple-comparison test by SigmaPlot. Scale bar, 1 cm. RSA, root system architecture; EBR, 24-epibrassinolide; DAT, days after transfer; PR, primary root; LR, lateral root.

As would be expected, *bri1-6* produced significantly shorter PR and LR and less number of LR as compared to WT (**Figures 6A–D**). Exogenous BR enhanced these root traits in WT across N conditions, but there was no effect on the PR length of *bri1-6* seedlings, although increases in LR number and length were seen under 11,400 µM and 500 µM N, respectively. More noteworthy is the observation that untreated or EBR-treated *bri1-6* showed a foraging response to mild N deficiency (550 µM) and an inhibitory response to severe N deficiency (10 µM) in relation to LR number and length, but not PR length (**Figures 6C, D**). These results indicate that either the weak allele *bri1-6* allows for some RSA changes or signaling pathways other than BR are responsible for the RSA changes in the *bri1-6* mutant.

### Exogenous BR and *bzr1-1D* enhance root meristem growth under low N levels

**Figure 7:**
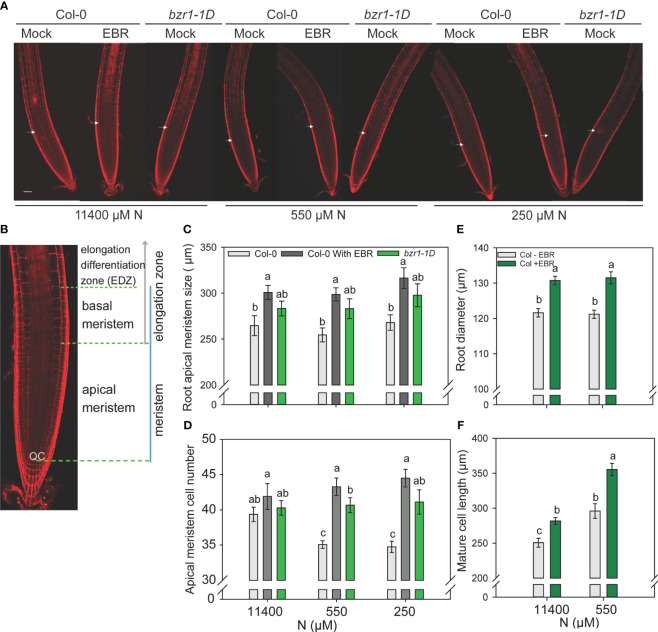
Confocal microscopy images of Col-0 (WT) and *bzr1-1D* root meristem. Seedlings grown in full N for three days were transferred to N sufficient (11400) and N deficient (550 and 250 μM N) medium supplemented with 0.05 nM EBR. Roots were stained with propidium iodide and visualized under confocal microscopy at four DAT. Representative root meristem images of Col-0 and bzr1-1D roots **(A)**, confocal image of Arabidopsis WT root meristem showing apical meristem [from the quiescent center (QC) to the first notable larger cortical cell] and basal meristem zone [starts from the end of apical meristem and ends at the start of elongation/differentiation zone (EDZ)] **(B)**, root apical meristem size **(C)**, root meristem cell number **(D)**, root diameter in mature zone **(E)** and mature cortical cell length. **(F)**. Bars represent means ± SEM (two independent biological replicates, *n*=15). Different letters indicate significant differences at *P* <0.05 according to two-way ANOVA and Tukey’s multiple comparison test by SigmaPlot. Scale bars, 50 μm. Arrow indicates the transition zone between apical and basal meristem.

In this study, 3-day-old WT and *bzr1-1D* seedlings grown on MS medium were transferred to and further grown on 11,440 µM (sufficient) and 550 µM and 250 µM (mild deficiency) N in the absence or presence of 0.05 nM EBR. Roots were stained with propidium iodide and visualized under confocal microscopy at 4 DAT (**Figures 7A, B**). There was no significant change in the apical meristem size of WT with decreasing N supply. However, exogenous EBR significantly increased the meristem size by 14%, 17%, and 18% at 11,400 µM, 550 µM, and 250 µM N, respectively, as compared to untreated control (**Figure 7C**). A similar pattern of increase in meristem size was also observed in *bzr1-1D* under all N conditions. Root meristem cell number in WT decreased by ~11% under mild N deficiency as compared to N-sufficient conditions. By contrast EBR not only maintained but also increased the meristem cell number by 10% and 13% at 550 µM and 250 µM N, respectively, as compared to N-sufficient condition (**Figure 7D**). There was no change in the meristem cell number in *bzr1-1D* under N-deficient conditions; however, the meristem cell number in EBR-treated and *bzr1-1D* remained considerably higher than that in untreated WT under N-deficient conditions. EBR increased the root diameter by 7% and 9% under N-sufficient and N-deficient conditions, respectively (**Figure 7E**). Exposure to low N increased the length of cortical cells in the root mature zone in WT, but EBR further increased cell length by 12% and 20% under 11,400 µM and 550 µM N conditions, respectively (**Figure 7F**). Thus, in *Arabidopsis*, exogenous EBR and constitutive activity of BZR1 positively impact meristem size, meristematic cell number, mature cortical cell length, and root diameter in the mature region under both N-sufficient and N-deficient conditions.

### *bzr1-1D* produced higher biomass than WT under N deficiency

**Figure 8:**
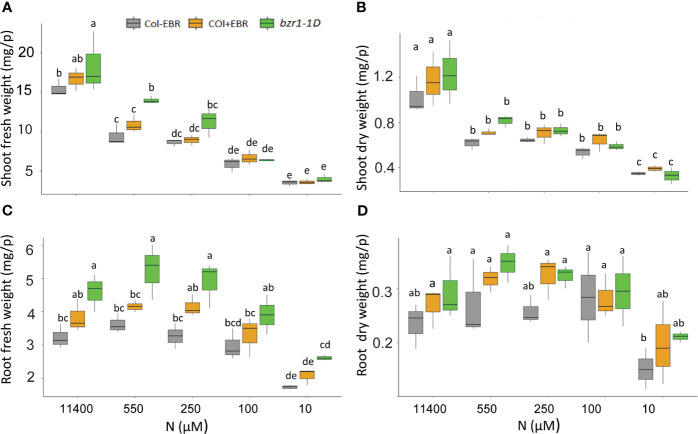
Fresh and dry shoot and root weights of untreated and BR-treated WT and *bzr1-1D* under different N conditions. Seedlings grown in full N for 3 days were transferred to N-sufficient (11,400 μM) and N-deficient (550 µM, 250 µM, 100 µM, and 10 µM N) media supplemented with 0.05 nM EBR. Root and shoot fresh weight were measured at 13 DAT. Shoot fresh weight **(A)**, shoot dry weight **(B)**, root fresh weight **(C)**, and root dry weight **(D)**. Bars represented means ± SEM (three independent biological replicates, *n* = 45). Different letters indicate significant differences at *p* < 0.05 according to two-way ANOVA and Tukey’s multiple-comparison test by SigmaPlot. BR, brassinosteroid; WT, wild type; EBR, 24-epibrassinolide.

Although RSA traits increased in response to mild N deficiency (**Figures 1**–**3**), the shoot fresh weight showed a consistent decline with increasing N stress, resulting in a reduction from 15.4 mg per plant at 11,400 N supply to 3.5 mg per plant at 10 µM N in untreated WT (**Figure 8A**). A statistically insignificant increase over WT, but with the same downward trend in response to N deficiency, was seen in the shoot weight of EBR-treated seedlings. Significant increases of 19% and 48% over WT were observed for shoot fresh weight in *bzr1-1D* at 11,400 µM and 550 µM N, respectively, which declined with increasing N deficiency (**Figure 8A**). There were no significant differences in the dry weight of shoots between WT (untreated and EBR-treated) and *bzr1-1D* (**Figure 8B**).

The general trend of RSA trait enhancement in EBR-treated and *bzr1-1D* seedlings over WT (**Figures 1**–**3**) was also reflected in root fresh and dry weights across N conditions (**Figures 8C, D**). *bzr1-1D* showed the greatest increase in root fresh weight over WT and EBR-treated seedlings while following the same trend as WT across N conditions. The increases in *bzr1-1D* were 43%, 45%, and 50% over WT at 11,400 µM, 550 µM, and 250 µM N, respectively, (**Figure 8C**). Due to the small size roots of seedlings, the dry weight changes were statistically insignificant but followed the same trend across N conditions (**Figure 8D**).

### *bzr1-1D* has higher N content under mild N deficiency

**Figure 9:**
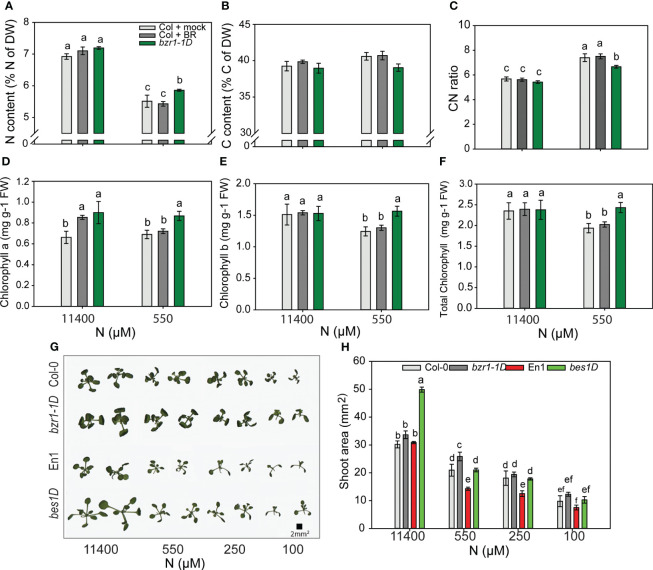
Morphophysiological traits of WT and gain-of-function BR mutants in response to declining N concentrations. **(A–C)** Nitrogen content **(A)**, carbon content **(B)**, and CN ratio **(C)**. Seedlings grown in full N for 3 days were transferred to N-sufficient (11,400 μM) and N-deficient (550 µM, 250 µM, 100 µM, and 10 µM N) media supplemented with 0.05 nM BR. Plants were harvested at 13 DAT, dried until constant weight, and used for C and N content measurements using the CN analyzer. **(D–F)** Chlorophyll *a* content **(D)**, chlorophyll *b* content **(E)**, and total chlorophyll content **(F)** of Col-0 and *bzr1-1D* plants grown as described above. Images of shoots of wild types (Col-0 and En-1) and gain-of-function BR mutants (*bzr1-1D* and *bes1-D*) were taken using EPSON scanner. Two plants represent each N condition **(G)** and shoot area **(H)**. Bars represent means ± SEM, average of three independent biological replicates. Different letters indicate significant differences at *p* < 0.05 according to two-way ANOVA and Tukey’s multiple-comparison test by SigmaPlot. WT, wild type; BR, brassinosteroid; DAT, days after transfer.

To see if BR-mediated RSA changes in response to N deficiency corresponded with higher N content, we analyzed % N of the dry weight of the shoot. Notwithstanding the increase in RSA traits under 550 µM (**Figure 2**), the N content in untreated WT decreased by 25% from sufficient N to mild N deficiency created by 550 µM. Exogenous BR had no effect, but *bzr1-1D* had 6% more N than WT at 550 µM (**Figure 9A**). There was no change in the C content in any of the seedlings (**Figure 9B**); consequently, the CN ratio increased from sufficient N to deficient N with *bzr1-1D* showing the least change (**Figure 9C**). *bzr1-1D* shoots accumulated higher levels of chlorophyll *a* (**Figure 9D**), chlorophyll *b* (**Figure 9E**), and total chlorophyll (**Figure 9F**) as compared to WT, particularly under mild N deficiency (550 µM N). While the chlorophyll *b* level declined for WT under N deficiency, it was maintained at the normal level in *bzr1-1D* (**Figures 9D–F**). EBR increased chlorophyll *a* level over WT only under full N.

To see if the decline in shoot fresh and dry weights and decrease in shoot N content with declining N supply (**Figures 8A, B**) were correlated with shoot size, the shoot area was calculated using ImageJ. Both Col-0 and En-1 (WTs) showed a steady decrease in shoot area with decreasing N (**Figure 9G**); e.g., there was 50% and 200% reduction in Col-0 under 550 µM and 10 µM N, respectively, as compared to 11,400 µM N (**Figure 9H**). *bzr1-1D* and *bes1-D* showed the same trend but exhibited higher shoot area across N conditions, with *bes1-D* showing significant increases over WT. At 550 µM N, *bzr1-1D* and *bes1-D* displayed 20% and 31% more shoot area than their respective WTs (**Figure 9H**). Collectively, these results indicate that shoot size decreases with increasing N deficiency, which correlates with the decrease in shoot biomass and shoot N content. Though not calculated, the estimated increase in root:shoot ratio under mild N conditions likely follows the optimal partitioning theory (McCarthy and Enquist, 2007; Kobe et al., 2010) where more biomass is partitioned to the roots and less to the shoots to maximize resource uptake.

### Quantification of relative gene expression in *bzr1-1D* roots under low N

**Figure 10:**
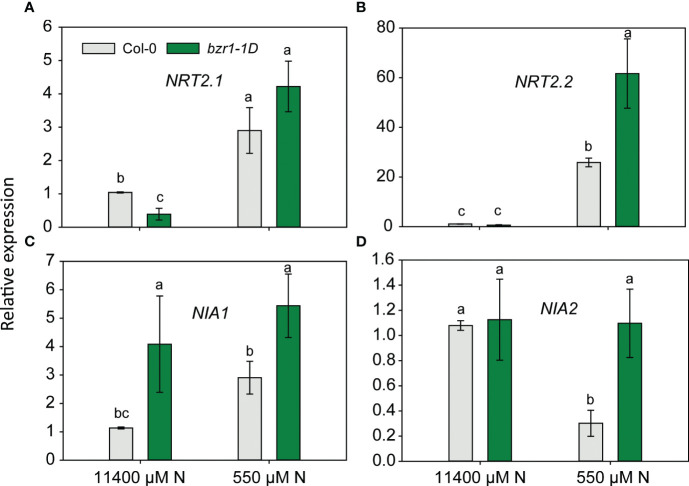
Relative expression of nitrogen transporter and assimilation genes in *bzr1-1D* roots under low N condition. Relative expression of *NRT2.1* and *NRT2.2* **(A, B)** and nitrate reductase genes *NIA1* and *NIA2* **(C, D)**. Seedlings grown in full N for 3 days were transferred to N-sufficient (11,400 μM) and N-deficient (550 µM N) media. Root tissues were harvested at 4 DAT. The expression levels of genes were measured using qRT-PCR and normalized to *U-box*. Bars represent means ± SEM, average of three independent biological replicates. Different letters indicate significant differences at *p* < 0.05 according to two-way ANOVA and Tukey’s multiple-comparison test by SigmaPlot. DAT, days after transfer.

Since EBR and constitutive activity of BZR1 enhanced RSA traits and the latter also enhanced the N content in the shoot of *Arabidopsis* seedlings (**Figures 3**, **9**), the expression of *NRT2.1* and *NRT2.2* and nitrate reductase genes *NIA1* and *NIA2* was quantified in WT and *bzr1-1D* roots under mild N deficiency. The expression of *NRT2.1* and *NRT2.2* was enhanced in both *WT* and *bzr1-1D* in response to N deficiency (**Figures 10A, B**), but only *NRT2.2* was expressed at 2.4-fold higher levels in *bzr1-1D* over WT under 550 µM N condition (**Figure 10B**). The expression of *NIA1* increased while that of *NIA2* decreased in WT in response to low N (**Figures 10C, D**). *bzr1-1D* expressed *NIA1* at ~2-fold higher level than WT under both N-sufficient and N-deficient conditions, while the expression *NIA2* was 3.5-fold higher than that in WT only under the N-deficient condition (**Figures 10C, D**). Thus, BR positively regulates the expression of these genes under N deficiency.

## Discussion

N deficiency leads to changes in growth and development, including those related to RSA (Crawford and Glass, 1998; Zhang et al., 1999; Remans et al., 2006). Since RSA is a complex trait, multiple signaling pathways are involved in its regulation under N deficiency (Jung and McCouch, 2013; Kiba and Krapp, 2016; Jia and von Wirén, 2020). Plant hormones function as key integrators of N signals to regulate root developmental programs (Kiba et al., 2011). BR controls many aspects of root development such as the root meristem size and LR development and elongation, but its role in nutrient deficiency responses has not yet been established (González-García et al., 2011; Hacham et al., 2011; Wei and Li, 2016; Li et al., 2020b). Since BR confers tolerance to a range of abiotic stresses (Divi and Krishna, 2009; Sahni et al., 2016), including stages of seedling development (Chakma et al., 2021); it is plausible that BR impacts changes in RSA in response to N deficiency that confer tolerance to N deficiency. Here, we show that BR modulates RSA under a range of N conditions and that *bzr1-1D* expresses enhanced RSA traits under these conditions, leading to better growth of *Arabidopsis* seedlings under N-deficient conditions.

### Exogenous BR enhances root foraging RSA traits in response to N deficiency

*Arabidopsis* seedlings exhibited a foraging response at mild N deficiencies generated by 550 µM and 250 µM N supply and a survival strategy at severe N deficiency generated by 10 µM N (**Figures 1**, **2**). In agreement with previous observations (Zhang et al., 1999; López-Bucio et al., 2003; Remans et al., 2006; Gruber et al., 2013), mild N deficiency increased PR and LR length and LR number with maximal impact seen as the total LR length per plant (**Figures 1**, **2**). Exogenous EBR further enhanced these foraging traits in response to N deficiency, but this effect was concentration-dependent, with 0.05 nM EBR being the most effective (**Figure 1**). EBR also enhanced these traits under N-sufficient supply and severe N deficiency with the exception of LR length under the latter condition. The contrasting effects of EBR on LR length under mild and severe N deficiency indicate that perhaps the well-characterized role of BR in cell elongation by increasing the expression of cell wall loosening enzymes such as xyloglucan endotransglucosylases (XETs) (Fry et al., 1992; Xu et al., 2020), and BR’s role in cell division and elongation through interconnected mechanisms with other hormones such as auxin (Oh et al., 2020), may be differentially regulated under the two conditions. Auxin has emerged as the lead hormone in controlling LR initiation. Recently, it was demonstrated that BR signaling increases local auxin biosynthesis leading to auxin accumulation in LR tips, which is responsible for LR elongation (Jia et al., 2021). While BR signaling is sufficient for promoting cell elongation via auxin under N deficiency, high levels of auxin reversibly inhibit BR signaling (Devi et al., 2022). Thus, the balance between BR and auxin in the LR, defined by the N condition, may be a key factor in determining the promotion or attenuation of LR growth.

Further evidence suggesting a vital role for BR in RSA modification under N deficiency derives from the work of Jia et al. (2019); Jia et al. (2020), who demonstrated that enhanced BR biosynthesis and signaling through increased expression of BR biosynthesis enzyme DWARF1 (DWF1) and BR INSENSITIVE1-ASSOCIATED KINASE1 (BAK1), respectively, are linked with PR elongation in response to low N. Jia et al. (2019) noted an increase in PR length in *bri1* mutants in response to low N, in addition to LR growth enhancement. We also found the *bri1-6* mutant, with a considerably reduced root system, to display a foraging response with an increase in LR number and length, but not in PR length, under mild N deficiency (**Figure 6**). There could be a few possibilities as to why *bri1* mutants respond to low N: 1) another pathway, possibly involving auxin, mediates the low N response in *bri1* mutants; 2) BES1/BZR1 are activated via a pathway other than BR (Shi et al., 2022); or 3) BR receptors other than BRI1, such root specific BRL1 and BRL3 (Caño-Delgado et al., 2004), mediate the response to N deficiency. Since *bri1-6* is a weak mutant allele, it is also possible that some BR signaling occurs via the mutant receptor.

### BR signaling through BZR1 and BES1 regulates RSA under N deficiency

The highly homologous transcription factors BZR1 and BES1 perform both redundant and unique roles in BR regulation of plant development (Wang et al., 2002; Yin et al., 2005). These transcription factors play a role in root meristem development (Salazar-Henao et al., 2016; Espinosa-Ruiz et al., 2017; Li et al., 2020a). The BZR1/BES1 module has also been implemented in adaptive responses of roots under low iron and phosphate (Singh et al., 2014, 2018). As a logical extension of our observations of EBR-mediated RSA modifications under N deficiency, we studied root responses in gain-of-function mutants *bzr1-1D* and *bes1-D*. Both mutants exhibited RSA traits similar to those of seedlings treated with EBR and under some conditions, e.g., severe N stress, an even stronger response than exogenous EBR (**Figure 2**). These results provide strong evidence for BR-mediated RSA modification under N deficiency to occur via the BZR1/BES1 module. That both *bes1-D* and *bzr1-1D* showed considerable LR elongation under severe N deficiency (10 µM N) (**Figure 3E**), where exogenous BR had little effect, suggests that the BES1/BZR1 module plays an important role in LR elongation. This is reminiscent of the role of BES1 in LR development through its regulation of *XTH19* and *XTH23* genes, which are involved in LR development under salt stress (Xu et al., 2020). *XTH19* and *XTH23* encode members of the XTH enzyme family, which participate in cell wall-related processes such as cell elongation and regulation of cell wall mechanical properties under stress (Xu et al., 2020).

The reduction of PR length in *bes1-D* as compared to its WT (**Figure 3B**) may be a mimic of supraoptimal exogenous BR concentrations that lead to inhibition of PR length (**Figure 1B**) (Müssig et al., 2003; González-García et al., 2011), presumably arising from excessive BES1-mediated transcription activity in *bes1-D*. However, the considerably higher LR number and LR length than WT in *bes1-D* either negate this assumption or suggest that mechanisms governing PR length are independent of those governing LR number and length. While mutants and exogenous BR are valuable tools to obtain an overall big picture, they are less likely to provide an accurate picture when a hormone functions in a gradient. BR signaling functions in a gradient to control meristem activity and timely cell elongation (Vukašinović et al., 2021).

Although both *bzr1-1D* and *bes1-D* were more resistant than WT to inhibition of RSA traits by the BR biosynthesis inhibitor BRZ, they nevertheless showed suppression of the RSA traits studied (**Figure 4**), indicating that optimal levels of endogenous BR are required to support the augmented expression of RSA traits in these mutants. This is not surprising considering that a single transcription factor is unlikely to be capable of covering the multitude of aspects of root development controlled by BR in collaboration with other signaling pathways. The BZR1/BES1 module acts as an integration hub of multiple signals that regulate plant growth and development (Li et al., 2014). As basic helix-loop-helix (bHLH)-type transcription factors, they interact with other proteins to coordinate growth and stress responses; however, crosstalk of BR with other hormones and signals also occurs via BR signaling components other than BZR1/BES1. For example, BIN2-mediated phosphorylation of ARFs (ARF7 and ARF19) leads to enhanced expression of *LATERAL ORGAN BOUNDARIES-DOMAIN16* (*LBD16*) and *LBD29* to regulate LR organogenesis (Cho et al., 2014). Some differences in the RSA traits of *bzr1-1D* and *bes1-D* and the response of the mutants to BRZ such as greater sensitivity to BRZ-mediated inhibition of LR number and length may reflect differences in the roles of these two transcription factors in controlling RSA.

### BR increases apical meristem size and meristematic cell number under low N

Root growth and development are determined by cell proliferation and expansion and cells attaining their final fate. Cell division is concentrated in the root meristem zone, from which cells exit and undergo expansion and differentiation. Periclinal divisions in the root meristem lead to its expansion in width and the addition of cells to the procambium in the stele (Beemster and Baskin, 1998). BR regulates both longitudinal and radial growth of the meristem by exerting an effect on cell shape and growth anisotropy (Fridman et al., 2021). The differential growth mediated by BR in the outer and inner cell layers shapes the meristem. We found exogenous EBR to increase the root apical meristem size under both N-sufficient and mild N-deficient conditions (**Figures 7A, C**). N deficiency reduced meristem size in WT, but not in *bzr1-1D* (**Figures 7A, C**). Exogenous EBR increased meristem cell number in both WT and *bzr1-1D* under N-deficient conditions (**Figure 7D**). These results are in agreement with the previously established role of BES1/BZR1 in stimulating QC division (Lee et al., 2015). Consistent with its role in cell elongation (Chaiwanon and Wang, 2015; Nolan et al., 2020), EBR also increased cell length under N-sufficient and N-deficient conditions.

### *bzr1-1D* displayed higher shoot and root biomass under both N-sufficient and N-deficient conditions

Plant biomass depends on cell expansion and cell proliferation, and BR regulates these processes to induce plant growth (Xie et al., 2011). BR stimulates cell expansion by increasing the expression of genes encoding XETs and pectin lyase-like enzymes (Uozu et al., 2000; Guo et al., 2009). Plants optimize biomass partitioning to maximize whole plant growth rate according to the external environment (Hilbert, 1990). Plants grown in N-deficient conditions increase their root biomass in order to increase the root volume for the uptake of more nutrients (Thornley, 1972; Wilson, 1988). The fact that EBR-treated and *bzr1-1D* displayed higher shoot and root biomass under both sufficient and low N conditions over WT (**Figure 8**) is in line with the role of BR in plant growth, including its ability to regulate cell expansion and cell proliferation and increase photosynthetic ability. EBR-mediated increase in meristematic cell number and cell length under N deficiency seen in this study (**Figure 7**) falls within the scope of BR’s role in enhancing growth. The noteworthy increase in root biomass under low N over sufficient N with a concomitant decrease in shoot biomass under the same conditions (**Figure 8**) is a clear indication of biomass partitioning in favor of root growth for better uptake of N. There is evidence indicating that BR regulates N uptake in plants (Sun et al., 2010; Zhao et al., 2016; Xuan et al., 2017; Xing et al., 2022). The higher N content in *bzr1-1D* is likely due to higher N uptake via the enhanced RSA components in this mutant under low N (**Figure 9A**). The higher expression of nitrogen transporter *NRT2.2* and nitrate reductase *NIA1* and *NIA2* in *bzr1-1D* also indicate that constitutive activity of BR transcription factors may help plant to uptake more N under deficient conditions. These results should encourage evaluation of BR pathway-associated genes in breeding programs for increasing N uptake and N use efficiency of crop plants.

## Conclusion

We provide comprehensive reference datasets of BR-mediated changes in RSA under N-sufficient and N-deficient regimes. This study demonstrated that BR enhances RSA components such as LR number and length under both N-sufficient and N-deficient conditions with the effect being greater under mild N deficiency as compared to N-sufficient condition. The gain-of-function BR mutants *bzr1-1D* and *bes1-D* undergo RSA changes under N deficiency, which is consistent with the results of exogenous BR applications. The *bzr1-1D* mutant displayed higher fresh and dry shoot and root weight, chlorophyll content, and higher N content than the WT when grown under mild N deficiency. Further studies are required to confirm that the higher N content in *bzr1-1D* is due to increased uptake of N by the roots. Overall, the results of this study suggest that BZR1 could be a promising target for molecular breeding programs aimed at enhancing RSA traits for better tolerance to N deficiency stress.

## Data availability statement

The original contributions presented in the study are included in the article/**Supplementary Material**. Further inquiries can be directed to the corresponding author.

## Author contributions

MA-M: Writing – original draft, Validation, Investigation, Formal analysis, Data curation. CC: Writing – review & editing, Supervision, Resources. PK: Writing – original draft, Resources, Writing – review & editing, Validation, Supervision, Project administration, Funding acquisition, Data curation, Conceptualization.
