# Supplementary material for: BZR1 and BES1 transcription factors mediate brassinosteroid control over root system architecture in response to nitrogen availability
Source: Front Plant Sci. 2024 May 8;15:1387321. doi: 10.3389/fpls.2024.1387321 (PMC11109456; doi:10.3389/fpls.2024.1387321)
Supplement: Supplementary file 1 [file Table_1.docx]

**Supplementary Table S1.** List of RT-qPCR primers used in this study

| **Gene** | **Primer** | **Sequence (5´→3´)** |
| --- | --- | --- |
| *NRT2.1* | F | CCGAGTACTTCTTTGACAGGTTT |
|  | R | CACACAGAAGAGGCCACCAG |
| *NRT2.2* | F | GCCAAAGACAAATTCGGAAAGATTC |
|  | R | CTGCTATAATCCCCGCTGTGT |
| *NIA1* | F | TTGACCACCAGGAGAAACCG |
|  | R | TCATCCCCATGAGGTTCCAGA |
| *NIA2* | F | CCACGTCCCTAAAGCCCAAT |
|  | R | TCTGTTCCTTACGGCGGTTC |
| *U Box* | F | TGCGCTGCCAGATAATACACTATT |
|  | R | TGCTGCCCAACATCAGGTT |

F: Forward; R: Reverse
